# Mitophagy and cancer

**DOI:** 10.1186/s40170-015-0130-8

**Published:** 2015-03-26

**Authors:** Aparajita H Chourasia, Michelle L Boland, Kay F Macleod

**Affiliations:** The Ben May Department for Cancer Research, The University of Chicago, 929 East 57th Street, Chicago, IL 60637 USA; The Committee on Cancer Biology, The University of Chicago, 929 East 57th Street, Chicago, IL 60637 USA; The Committee on Molecular Metabolism & Nutrition, 929 East 57th Street, Chicago, IL 60637 USA; The Ben May Department for Cancer Research, The University of Chicago Comprehensive Cancer Center, The Gordon Center for Integrative Sciences, W338 929 East 57th Street, Chicago, IL 60637 USA

**Keywords:** Mitophagy, Autophagosomes, Parkin, BNIP3, NIX, Mitochondrial dysfunction

## Abstract

Mitophagy is a selective form of macro-autophagy in which mitochondria are selectively targeted for degradation in autophagolysosomes. Mitophagy can have the beneficial effect of eliminating old and/or damaged mitochondria, thus maintaining the integrity of the mitochondrial pool. However, mitophagy is not only limited to the turnover of dysfunctional mitochondria but also promotes reduction of overall mitochondrial mass in response to certain stresses, such as hypoxia and nutrient starvation. This prevents generation of reactive oxygen species and conserves valuable nutrients (such as oxygen) from being consumed inefficiently, thereby promoting cellular survival under conditions of energetic stress. The failure to properly modulate mitochondrial turnover in response to oncogenic stresses has been implicated both positively and negatively in tumorigenesis, while the potential of targeting mitophagy specifically as opposed to autophagy in general as a therapeutic strategy remains to be explored. The challenges and opportunities that come with our heightened understanding of the role of mitophagy in cancer are reviewed here.

## Mitophagy: the major players

Macro-autophagy (henceforth referred to as autophagy) is a highly conserved self-degradative process by which cytosolic constituents, including organelles, protein aggregates, and pathogens are captured by nascent phagophore membranes and degraded through fusion of the resulting autophagosomes with lysosomes [1,2]. As such, autophagy plays an important housekeeping function for the cell in getting rid of large and potentially toxic structures [1,3-6]. Autophagy also plays a critical role in recycling the breakdown products generated in the form of amino acids, nucleic acids, fatty acids, and ATP that are released by the lysosome and used in the cell to maintain metabolism, growth, and survival under conditions of nutrient deprivation [7].

There are bulk degradative forms of autophagy that are largely non-selective for cytosolic cargo, as well as targeted autophagy that selectively engulfs and degrades specific cargoes [8-13]. Mitophagy is a classic example of the latter that involves the selective targeting of mitochondria for degradation at the autophagosome through interactions of key adaptor molecules at the outer mitochondrial membrane (OMM) with processed LC3 (or related molecules) at the growing phagophore membrane [8,14-16]. These adaptor molecules include BNIP3, NIX, and FUNDC1 in addition to mitochondrial targets of E3 ubiquitin ligases functioning at the mitochondria, such as Parkin and Mul1, as will be discussed below.

Mitophagy promotes turnover of dysfunctional mitochondria that would otherwise damage the cell, but how the cell distinguishes between functional and non-functional mitochondria is not entirely elucidated. Loss of mitochondrial membrane potential and mitochondrial fragmentation precede mitophagy [17-19], suggesting that this plays a role in their selective uptake by autophagosomes. Indeed, mitochondrial depolarization plays a direct role in activating Parkin-dependent mitophagy by inducing PINK1 kinase stabilization at the OMM [20-22]. Mitochondrial membrane depolarization also induces proteolytic cleavage and degradation of the fusion protein Opa-1 thereby reducing the size of mitochondria, a consequence that is likely to favor uptake of mitochondria by phagophore membranes while also linking mitochondrial turnover to loss of function [23,24]. Conversely, mitochondrial fusion protects healthy respiring mitochondria from degradation, a mechanism that is promoted by protein kinase A (PKA)-mediated inhibition of the fission protein Drp-1 in response to nutrient deprivation, for example [18,19].

The accumulation of dysfunctional mitochondria with time contributes to the aging process likely due to accumulation of reactive oxygen species (ROS)-induced mtDNA mutations in line with the ‘free radical theory of aging’ since mouse life span can be increased and age-related phenotypes can be ameliorated through over-expression of mitochondrial catalase [25,26]. However, mitophagy also plays a key role in reducing mitochondrial mass in the acute response to certain stresses, such as hypoxia and nutrient deprivation [16,27-29]. This involves the turnover of otherwise healthy mitochondria, but it is not clear to what extent healthy mitochondria are rendered dysfunctional by stress-induced signaling molecules and if this requires the active involvement of some or all of these signaling molecules in mitochondrial membrane depolarization and fragmentation of healthy mitochondria.

Dissection of the functions of some of the regulators and molecular adaptors involved in targeting mitochondria to the autophagosome has increased our understanding of how mitophagy is initiated and executed. The most extensively characterized of these mitophagy regulators are Parkin and Pink1, as well as BNIP3 and NIX that play distinct and non-overlapping activities to promote mitophagy [30-32]. While this current cast of mitophagy-specific modulators is rather limited, it is clear that additional players (such as Mul1 and FUNDC1) are emerging and likely to be the focus of future studies. Here, we first review current knowledge of molecular regulators of mitophagy with recognized roles in tumorigenesis.

## Parkin and PINK1

The *PARK2* (Parkin) and *PARK6* (PINK1) gene products were originally identified as mutated in human Parkinson’s disease (PD) and subsequently shown to function in concert to promote mitophagy, thus implicating dysfunctional mitochondria in the etiology of PD [15]. *PARK2* (Parkin) maps to a common fragile site at human chromosome 6q25-q26 that is frequently deleted in ovarian, breast, bladder, lung, and other cancers [33,34]. Consistent with a tumor suppressor function for Parkin, *parkin* null mice are susceptible to spontaneous liver tumors [35] that may be linked to functions of Parkin in lipid metabolism in the liver [36]. Parkin null mice are also sensitized to irradiation-induced lymphomagenesis [37]. Parkin expression increased oxidative metabolism and limited the Warburg effect downstream of the p53 tumor suppressor, most likely by enhancing mitochondrial integrity, possibly explaining the tumor suppressive activity of Parkin [37]. As a component of the FBX4 Cullin-ring ligase complex, Parkin has also been shown to regulate levels of Cyclin D1, Cyclin E, and CDK4 in cancers [34], suggesting that in addition to its role in mitophagy, Parkin may also elicit its tumor suppressor functions through inhibition of the cell cycle.

**Figure 1:**
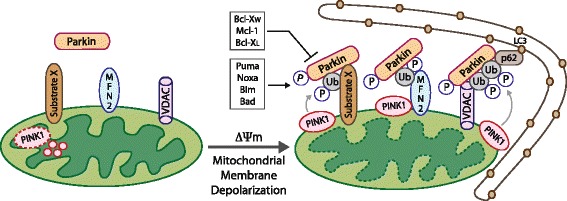
**Parkin recruitment to depolarized mitochondria promotes their degradation by mitophagy.** In polarized mitochondria, PINK1 is degraded in the mitochondrial matrix (left), but upon membrane depolarization, PINK1 is stabilized and accumulates at the OMM, where it phosphorylates Mfn-2 and other substrates, including ubiquitin, that act as receptors for Parkin. Once Parkin is recruited to the OMM, it ubiquitinates key protein substrates including VDAC1 and Mfn-2, and other possibly unknown targets (substrate X). Parkin-dependent ubiquitination of VDAC1 and other mitochondrial proteins promotes interaction with p62/Sqstm1 that in turn facilitates interaction with LC3 at nascent phagophores thereby targeting depolarized mitochondria for degradation by autophagy.

The localization of the Parkin E3 ubiquitin ligase to the mitochondria is regulated by the PINK1 (PTEN-induced putative kinase 1) serine/threonine kinase that undergoes voltage-dependent import leading to proteolysis at the inner mitochondrial membrane in healthy mitochondria but accumulates at the outer mitochondrial membrane in response to mitochondrial depolarization [20,21,22,38] (Figure 1). PINK1 phosphorylates Parkin directly but mutation of all serine and threonine residues in Parkin did not block its translocation to the mitochondria [39], and recent evidence shows that PINK1 phosphorylation of ubiquitin on serine 65 is required to recruit Parkin to mitochondria [39,40]. A large number of mitochondrial proteins have been identified as Parkin substrates at the OMM, including Vdac1, Miro, and Mfn-2 [15,41-43], and indeed systematic identification of all Parkin substrates indicates that the mitochondrial proteome is markedly altered by Parkin activity [43]. Specific targets such as Mfn-2 are phosphorylated by PINK1 at the OMM, and Mfn-2 has been shown to selectively recruit Parkin to damaged mitochondria [44]. However, the wide range of mitochondrial substrates that are ubiquitinated and then phosphorylated by PINK1 suggests that Mfn-2 may be only one of many receptors for Parkin at the mitochondria [43,39]. Furthermore, targeting of mitochondrial substrates by Parkin is highly dynamic [43] with the role of mitochondrial deubiquitinases such as USP30 in antagonizing Parkin-dependent mitophagy recently emerging [45] and suggesting that additional signaling inputs modulate Parkin’s role in mitophagy in response to stress.

Once ubiquitinated by Parkin, some of these substrates (such as ubiquitinated Vdac1) create a docking site for the LC3 interacting proteins p62/SQSTM1 and NBR-1 [46-48], allowing for selective Parkin-dependent degradation of mitochondria at the autophagosome (Figure 1). Recruitment of Parkin to depolarized membranes is inhibited by the anti-apoptotic Bcl-X_L_, Mcl-1, and Bcl-W proteins in a Beclin-independent manner, although not by Bcl-2 itself [32]. Inhibition of mitophagy by Bcl-X_L_, Mcl-1, and Bcl-W involved their direct interaction with Parkin, blocking the interaction of Parkin with PINK1 and thus preventing the Parkin-dependent ubiquitination of mitochondrial targets [32]. Conversely, the pro-apoptotic BH3 proteins Puma, Noxa, Bim, and Bad, but not the non-canonical BH3 proteins BNIP3, Nix, or Beclin1, all promoted Parkin translocation to mitochondria, possibly by reducing the interaction of Parkin with the aforementioned Bcl-2-related molecules [32].

Alternative models to explain the role of Parkin in mitophagy have also been proposed in which Parkin acts much more indirectly. This speculation about how Parkin promotes mitophagy has arisen due to the growing appreciation that no single Parkin substrate is essential for mitophagy [49] and that several Parkin substrates are degraded by the ubiquitin-proteasome system independent of autophagy [42]. One particularly intriguing alternative explanation for the function of Parkin in mitophagy emerges from evidence that targeted proteasomal degradation of Parkin substrates imbalances the ratio of mitochondrial to nuclear encoded proteins at the mitochondria, resulting in the mitochondrial unfolded protein response (UPR^mt^) [50]. The UPR^mt^ renders mitochondria dysfunctional and activates stress signaling that can result in mitophagy [16]. Alternatively, Parkin may promote mitophagy indirectly by inhibiting fusion (as a result of Mfn-1/Mfn-2 degradation) or by promoting degradation of an unknown mitophagy inhibitor at the mitochondria [15,42].

Regulation of mitochondrial transport along microtubules (MTs) is another key consequence of Parkin recruitment to mitochondria [22,49]. This is achieved through Parkin-mediated turnover of Miro, a protein that tethers MT-associated kinesin motor protein complexes to the OMM [41] and through Parkin-dependent recruitment of HDAC6 (a ubiquitin-binding protein deacetylase) that also promotes trafficking of mitochondria along MTs [46,51]. Clearly, regulation of mitochondrial trafficking by both Miro and HDAC6 is likely to be important for successful targeting of mitochondria to autophagosomes but again points to a more complex role for Parkin in mitophagy than was initially envisioned. Finally, Parkin has non-mitochondrial substrates that influence mitochondrial mass in cells, such as the PARIS transcriptional regulator that represses PGC-1α expression to inhibit mitochondrial biogenesis [52].

## BNIP3 and NIX

Mitophagy has emerged as a key adaptive response to hypoxia, as cells attempt to reduce their mitochondrial mass to not only limit ROS production but also maximize the efficient use of available oxygen [16]. Two key molecular mediators implicated in promoting hypoxia-induced mitophagy are BNIP3 and NIX (also known as BNIP3L) [31,48,53]. Both are target genes of the hypoxia-inducible factors (HIFs) [54,55] although BNIP3 is more rapidly induced and to higher levels than NIX as oxygen levels drop due to the differential dependence of BNIP3 and NIX mRNA expression on the two transactivation domains in HIF-1α [56-58]. BNIP3 is also transcriptionally regulated by RB/E2Fs [28], NF-κB [59], FoxO3 [60], oncogenic Ras [61,62], and p53 [63], while NIX is regulated by p53 [64]. They both also exhibit distinct tissue-specific patterns of expression with BNIP3 most strongly expressed in the heart, liver, and muscle while NIX is expressed strongly in hematopoietic tissues and testes [65,66]. Consistently, NIX plays a key developmental role in red blood cell maturation promoting mitochondrial clearance from maturing reticulocytes [67,68], while BNIP3 is involved in modulating mitochondrial integrity in the skeletal muscle and liver [60,66].

**Figure 2:**
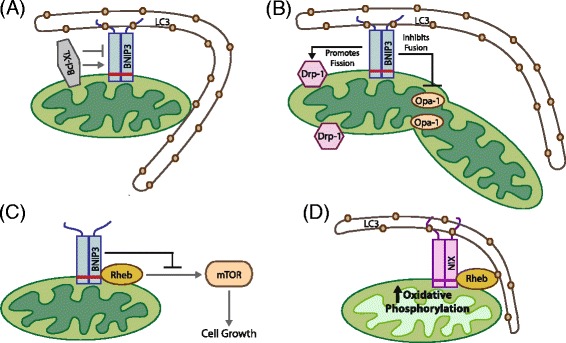
**BNIP/NIX promotes mitophagy through direct interaction with LC3 at the phagophore.** BNIP3 and NIX are both hypoxia-inducible genes that encode molecular adaptors that promote mitophagy through interaction with processed LC3-related molecules at nascent phagophores **(A)**. Both BNIP3 and NIX interact with Bcl-2 and Bcl-XL through their amino terminal ends, and Bcl-2/Bcl-XL has been postulated to play both positive and negative regulatory effects on BNIP3 function (A). BNip3 has also been shown to interact with regulators of mitochondrial fission (Drp-1) and mitochondrial fusion (Opa-1). These interactions are positive and negative, respectively, resulting in a role for BNIP3 in promoting fission while inhibiting fusion **(B)**. BNIP3 has also been shown to interact with the small GTPase, Rheb, resulting in reduced Rheb activity, reduced mTOR activity, and reduced cell growth **(C)**. This function for BNIP3 in modulating Rheb **(C)** contrasts with the proposed functional interaction of NIX with Rheb **(D)** that elicits a mTOR-independent effect on mitophagy by promoting LC3 processing and increased mitochondrial turnover in cells grown on oxidative substrates **(D)**. NIX is required for recruitment of Rheb to mitochondria and its activating effect on mitophagy.

BNIP3 and NIX integrate into the OMM as redox-resistant homo-dimers with a short 10 to 11 amino acid carboxy terminal tail in the intermembrane space and a proximal 23 amino acid transmembrane domain containing a critical glycine zipper that is required for both dimerization and membrane integration [69-71]. The remaining amino terminal portion of both BNIP3 and NIX protrudes out into the cytosol where both BNIP3 and NIX interact with LC3-related molecules at associated phagophore membranes [72,73] (Figure 2A). The direct interaction of BNIP3 and NIX with processed LC3B-II or GABARAP is dependent on a LC3-interacting region (LIR) located within an unstructured amino terminal region of each protein (amino acids 15 to 21 in BNIP3 and 43 to 49 in NIX) [72-74], and thus, similar to ATG32 in yeast [75,76], BNIP3 and NIX function to target mitochondria directly to the autophagosome for degradation. Binding of BNIP3 to LC3 is regulated by phosphorylation on serine residues adjacent to the LIR motif, but the identity of the kinases responsible is not known [77]. It remains to be determined to what extent other events, such as elevated ROS, membrane depolarization, or indeed altered electron flux at the respiratory chain, modulate the BNIP3/NIX structure to induce interactions with LC3 or other proteins involved in mitophagy.

Expression of both BNIP3 and NIX has been linked to non-apoptotic cell death in response to various stresses, and both proteins used to be categorized as BH3-only proteins [31]. However, more recent work has shown that the BH3 domain in both BNIP3 and NIX is weakly conserved and redundant for function [78,79]. Furthermore, various normal tissues express these proteins at high levels without inducing cell death [65,66], and thus, additional signals that either modify or disrupt BNIP3/NIX function are likely required for these proteins to induce cell death [29,80]. Thus, although linked to non-apoptotic cell death in early publications, the growing consensus is that BNIP3 and NIX function normally as mitochondria-specific receptors/cargo adaptors targeting mitochondria for degradation by autophagy and that it is disruption or inhibition of their function that leads to non-apoptotic cell death, although key aspects of this perspective remain to be formally tested experimentally.

Although not *bona fide* BH3 proteins, both BNIP3 and NIX do interact with Bcl-2 and Bcl-X_L_ through their amino terminal 49 amino acids [78], the region of both proteins that also mediates interaction with LC3-related molecules. Thus, it has been proposed that BNIP3/NIX interactions with Bcl-2 or Bcl-X_L_ can modulate binding of BNIP3/NIX to LC3 [77] (Figure 2A) although this has not been explored in a physiological context.

BNIP3-dependent mitophagy is preceded by mitochondrial fragmentation and perinuclear clustering of mitochondria [28,81]. Over-expression of exogenous BNIP3 induces mitochondrial fragmentation possibly due to the inhibitory interaction of BNIP3 with the fusion protein Opa-1, resulting in disruption of Opa-1 complexes and cristae remodeling [82,83] (Figure 2B). BNIP3 also induces translocation of the fission protein Drp-1 to mitochondria such that over-expression of either Mfn-1 or dominant negative Drp-1 inhibited BNIP3-dependent mitophagy [84] (Figure 2B).Thus, similar to Parkin and other signals that promote mitophagy, there is an intimate link between BNIP3 and regulators of mitochondrial fission and fusion, implicating modulation of mitochondrial dynamics in BNIP3-dependent mitophagy. Intriguingly, the ability of BNIP3 to promote mitochondrial fragmentation can be uncoupled from its ability to induce mitophagy, but again, the signals regulating the uncoupling of BNIP3 functions in mitochondrial fragmentation from its ability to promote mitophagy are not known.

Both BNIP3 and NIX also interact with Rheb, a small GTPase that acts positively upstream of mTOR to promote cell growth [74,85]. Rheb interacts with BNIP3 in a manner dependent on the transmembrane domain of BNIP3 consistent with Rheb only interacting with BNIP3 dimers at the OMM [85] (Figure 2C). Similar to the binding of Bcl-2 and Bcl-X_L_ to BNIP3 [78], Rheb binding also required the 30 amino terminal residues of BNIP3 [85], suggesting that Bcl-2 and Bcl-X_L_ may modulate the BNIP3-Rheb interaction. This work also reported that BNIP3 repressed Rheb activity resulting in reduced mTOR activity and slower cell growth [85], consistent with a tumor suppressor function for BNIP3.

By contrast, the interaction of NIX with Rheb elicited mTOR-*independent* effects on cell growth [74]. Rheb was recruited to the OMM under growth conditions that stimulated high levels of oxidative phosphorylation where Rheb interacted directly with NIX and processed LC3 (Figure 2D). Over-expression of Rheb promoted LC3 processing and increased mitophagy independent of mTOR activity but in a NIX-dependent fashion [74]. Thus, NIX appears to play a key role in recruiting Rheb to mitochondria under conditions of high oxidative phosphorylation leading to increased mitophagy that would be required to maintain a healthy pool of mitochondria under high rates of oxidative metabolism. Arguably, this more recent report identifying positive regulation of Rheb by NIX contrasts with the previous study in which BNIP3 repressed Rheb activity [85]. Clearly, NIX may function differently from BNIP3 with respect to Rheb activity in mitophagy, and further work will be needed to reconcile these findings.

BNIP3 and NIX have both been shown to be up-regulated in ductal carcinoma *in situ* (DCIS) in human breast cancer [86,87], while loss of BNIP3 expression at both the RNA and protein level in progression to invasive ductal carcinoma of the breast was associated with increased proliferative index and lymph node metastases [88]. In other cancers, including hematological malignancies and lung, gastric, pancreatic, and liver cancer, epigenetic silencing of BNIP3 expression as tumors progress to invasiveness and metastasis has been reported [89-92]. In pancreatic cancer in particular, inactivation of BNIP3 was associated with chemoresistance and a poor prognosis [89,93,94]. However, epigenetic silencing is not the likely mechanism of BNIP3 silencing in human breast cancer [95]. Interestingly, Tumorscape™ (Broad Institute, Cambridge, MA, USA) showed significant deletion around the BNIP3 locus at 10q26.3 in 7 out of 14 human tumor types, including breast cancer [96] while altered sub-cellular localization of BNIP3 in glioma, breast, and prostate cancer has also been reported [88,97-99]. Consistently, BNIP3 knockdown in the 4T07 orthotopic mammary tumor model promoted tumor growth and metastasis [100]. Tumor suppressor functions have also been attributed to NIX [64] although the relative importance of NIX in early-stage versus late-stage tumorigenesis has not been dissected. Thus, similar to Parkin [37], BNIP3 and NIX both appear to play tumor suppressor roles.

## Other mitophagy regulators

Mitochondrial uncoupling agents can rescue mitophagy defects in Nix null erythroblasts [68], indicating that alternative mitophagy mechanisms can be activated to promote mitophagy when one particular pathway is inactivated. Currently, there is no evidence to suggest that either BNIP3 or NIX requires Parkin activity to promote mitophagy. Conversely, while one report suggests that BNIP3 and NIX promote Parkin recruitment to mitochondria [101], another report indicates that they do not [32]. Redundancy between mechanisms of mitophagy would explain the lack of more severe phenotypes in mice genetically deleted for Parkin, BNIP3, or NIX [36,65,102]. Indeed, there are mitochondrial E3 ubiquitin ligase complexes other than Parkin involved in regulating mitophagy, such as Mul1, which is induced by FoxO1 and FoxO3 transcription factors in response to serum starvation and other stresses [103]. Mul1 promotes mitophagy in skeletal muscle, and this involves its ubiquitinating and targeting Mfn-2 for degradation, resulting in increased mitochondrial fission and mitophagy [103]. Another novel mitophagy mechanism involves the hypoxia-induced interaction of FUNDC1 protein at the OMM with LC3 at the phagophore through a conserved LIR motif in FUNDC1 [104]. Similar to the autophagy adaptor molecule NBR1, there is a tyrosine residue rather than the more common tryptophan at the critical +1 position in the LIR motif of FUNDC1 [104]. Intriguingly, this renders the FUNDC1-LC3 interaction subject to negative regulation by oncogenic SRC1 kinase activity that phosphorylates FUNDC1 at Y18 [104,105]. Conversely, phosphorylation of FUNDC1 by ULK-1 on serine 17, immediately adjacent to Y18 in the LIR motif of FUNDC1, promotes the interaction of FUNDC1 with LC3 and facilitates mitochondrial turnover [105]. ULK-1 translocation to mitochondria was induced by hypoxia (or mitochondrial uncoupling agents) where it was shown to interact directly with FUNDC1 [105]. Interestingly, FUNDC1 and NIX are both repressed by a hypoxia-induced microRNA, miR-137, thereby limiting the extent of mitophagy under hypoxia [106]. In summary, it is clear that there are multiple redundant pathways modulating mitochondrial turnover at the autophagosome and the key question remains how these mechanisms are coordinately regulated in response to different stresses and how they may be disrupted in cancer.

## Effects of autophagy inhibition versus mitophagy inhibition on tumorigenesis

Several recent publications have highlighted the accumulation of defective mitochondria as explaining the block to tumor progression when macro-autophagy is inhibited [107-110]. In most of these mouse tumor models, macro-autophagy was inhibited genetically through targeted deletion of either Atg5 or Atg7 in the context of K-Ras-driven oncogenesis [107-109,111]. While loss of autophagy promoted early growth of tumors, progression to late-stage and invasive disease was blocked highlighting a dual role for autophagy in cancer - tumor suppressive early, while tumor-promoting later. Based on these studies, it was proposed that Ras-driven tumors were ‘autophagy addicted’ [107] such that tumors expressing activated *K-Ras* depend on autophagy to maintain metabolic sufficiency under nutrient depletion, ischemia, or matrix detachment and this is particularly important at later stages of tumorigenesis [107-110].

In-depth analyses of autophagy-deficient tumors in these mice revealed the presence of clearly dysfunctional mitochondria that exhibited altered morphology, ineffective fatty acid oxidation, reduced carbon flux through Krebs cycle, and lipid accumulation [107-110]. This in turn was linked to increased glucose uptake and reduced oxygen consumption under aerobic conditions, both key features of the Warburg effect. Given these mitochondrial inefficiencies and the failure to progress to malignancy, it was suggested that these autophagy-deficient tumors were akin to oncocytomas [108], benign tumors forming in key endocrine organs that possess large numbers of swollen and dysfunctional mitochondria for as yet unexplained reasons [112].

What is not clear from these studies is the extent to which other defects arising from defective autophagy contribute to the altered tumor phenotype and failure of autophagy-deficient tumors to progress to malignant carcinoma. Critically, autophagy is required for amino acid recycling from the lysosome that plays a critical part in growth under conditions of nutrient deprivation, such as in ischemic tumors [1,5,113]. This could clearly contribute to the tumor phenotype in addition to the observed defects in mitochondria. Additionally, autophagy plays a key role in other processes that affect malignant progression, including elimination of unfolded proteins and reducing ER stress [114], effects on recruitment of tumor-associated immune cells and anti-tumor immunosurveillance [111,114-116], and secretion of cytokines and MMPs [117]. Thus, while there are clearly mitochondrial defects in tumors arising in mice deficient for autophagy as a whole, the overall tumor phenotype cannot currently be attributed entirely to the accumulation of defective mitochondria. This becomes particularly apparent when the effects of mitophagy deficiency on tumorigenesis are examined (Table 1). Loss of Parkin, as already mentioned, promotes the Warburg Effect, tumorigenesis in the liver, and irradiation-induced lymphomagenesis [35,37] while inhibition of BNIP3 or NIX promotes tumor progression [64,100]. Thus, based on currently available data, it appears that inhibition of mitophagy promotes tumor progression and does not phenocopy inhibition of autophagy, which blocks tumor progression (Table 1).

**Table 1:** **Comparison of the tumor phenotypes associated with deregulation of key regulators of mitophagy and general autophagy**

**Gene (human/mouse)**	**Linked human cancer**	**Mouse model phenotype**	**Reference**
Mitophagy regulators			
*Parkin*	*Parkin* located at chr. 6q25-q26 is significantly deleted in bladder, lung, breast, and ovarian cancer.	*Parkin* null mice are susceptible to liver tumors; sensitized to irradiation-induced lymphomagenesis.	[34,35,37]
*BNIP3/Bnip3*	*BNIP3* is up-regulated in DCIS; epigenetically silenced in hematologic, liver, lung, colorectal, and pancreas cancer.	Knockdown of BNIP3 promotes metastasis in an orthotopic mouse model of breast cancer.	[86-91,100]
*BNIP3L/Bnip3L (NIX/Nix)*	*NIX* is up-regulated in DCIS correlating with hypoxia.	Knockdown of NIX promotes tumorigenicity in xenograft model.	[64,86,87]
Autophagy regulators			
*BECN1/Becn1*	Mono-allelic deletion BECN1 in breast, ovarian, and prostate cancer, although linkage to BRCA1 calls significance of BECN1 deletion into question.	Becn1 heterozygotes are predisposed to lymphoma, hepatocellular carcinoma, and other cancers; Becln1 deficiency promotes tumor growth in xenografts.	[124-127]
*p62/SQSTM1*	p62/SQSTM1 amplification on chr.5q linked to clear cell renal cell carcinoma. Over-expressed in lung, breast and prostate cancer.	p62-null mice resistant to Ras-driven lung tumorigenesis. Loss of p62 reduces liver tumorigenesis in Atg7-deficient mice and other models.	[128-130]
*ATG5/Atg5*	Not reported.	Deletion of Atg5 promotes early-stage tumor growth in K-Ras driven lung and pancreas cancers but inhibits progression to malignancy.	[109,111]
*ATG7/Atg7*	Silenced in HNSCC.	Deletion of Atg7 promotes early-stage tumor growth in K-Ras and B-Raf-driven lung and K-Ras-driven pancreas cancers but inhibits progression to malignancy.	[108-110,131]
*FIP200 (RB1CC1)*	Inactivating truncation mutations found in human breast cancers leads to repression of the RB tumor suppressor.	Loss of FIP200 inhibits primary tumor growth and metastasis in the MMTV-PyVT mouse model of mammary tumorigenesis in a p62-dependent manner.	[116,132]

## Targeting mitophagy as an approach to adjuvant chemotherapy?

**Figure 3:**
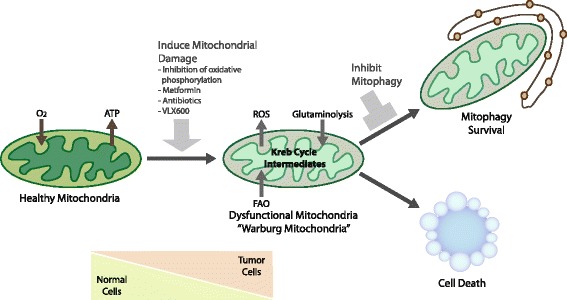
**Strategies to target mitophagy for cancer therapy.** Tumor cells are likely to be more dependent on functional mitophagy than normal cells due the increased requirement to manage ROS levels, due to dependence on key aspects of mitochondrial metabolism, such as glutaminolysis, particularly given the ischemic nature of advanced macroscopic tumors. Such a dependence on mitophagy could be exploited therapeutically by the development of specific small molecule inhibitors of mitophagy that could be combined with other drugs that induce mitochondrial dysfunction, such as respiratory inhibitors or antibiotics, to further increase the requirement for functional mitophagy.

The adverse tumor-promoting effects of chronic mitophagy inhibition arising from deletion or inactivation of genes such as Parkin and BNip3, particularly induction of the Warburg effect, argue against targeting mitophagy as a therapeutic strategy. However, for advanced tumors that have already undergone the switch to glycolytic metabolism but remain dependent on mitochondria for other metabolic functions, such as glutaminolysis, fatty acid oxidation, and generation of critical Krebs cycle intermediates, acute chemical inhibition of mitophagy remains a valid approach to be tested therapeutically. Since tumor cells already produce increased ROS compared to normal cells [118], the combined effect of further increased ROS and reduced mitochondrial metabolism arising from inhibition of mitophagy may be synergistic and promote efficient tumor cell killing while sparing normal cells that are less likely to have dysfunctional mitochondria and therefore likely to be less sensitive to mitophagy inhibition (Figure 3). Before such approaches can be adopted though, it will be necessary to investigate further how much mitochondrial damage or dysfunction can be tolerated by normal versus tumor cells, and for how long, before loss of viability. Once mitophagy is inhibited, for example, it is not clear how rapidly damaged mitochondria accumulate and to what extent this varies depending on cell type, the specific type of mitochondrial damage sustained, the nature of the damaging stress applied, or indeed the ability of the cell to adapt to mitochondrial dysfunction in other ways. For example, increased mitochondrial fusion may allow some cell types to distribute damaged mitochondrial content in such a way that cells can survive mitophagy inhibition. It will also be important to identify which tumors retain the capacity to undergo functional mitophagy and have not undergone selection for mitophagy inactivation through deletion of Parkin, or silencing of BNIP3, for example.

To overcome some of these potential caveats, complementary approaches combining acute mitophagy inhibition with drugs that inhibit glycolysis (to prevent the Warburg effect) might be more effective. Alternatively, acute induction of mitochondrial dysfunction could be used to unmask a dependence on mitophagy, as opposed to relying on mitophagy inhibition on its own that will only kill cells that have an inherently high normal rate of mitochondrial turnover and/or high rate of mitochondrial damage accumulation (Figure 3). Such acute stresses could include inhibiting respiration with metformin or other respiratory inhibitors. Interestingly, the increased sensitivity of K-Ras^G12D^; Lkb1 null lung tumors to phenformin (a more potent analog of metformin) was partially attributed to mitophagy defects in the absence of AMPK/ULK1 signaling downstream of Lkb1 [119]. Moreover, an RNAi screen to identify genes that sensitized tumor cells to low glucose found that inhibition of components of the electron transport chain was most effective in limiting the growth of patient-derived tumor cells [120]. This implicates mitochondrial oxidative phosphorylation taking place at the mitochondria as the key determinant of sensitivity to low glucose, providing further rationale for the use of biguanides, such as metformin, in cancer therapy [120]. Along similar lines, another recent study identified VLX600 as a drug that inhibits mitochondrial respiration, induces mitochondrial dysfunction, and preferentially kills tumor cells when exposed to nutrient stress [121]. In addition, the resistance of dormant tumor cells in K-Ras-driven pancreatic cancer to oncogene ablation was shown to be dependent on functional OXPHOS [122]. More speculatively, antibiotics such as tetracycline could be re-purposed for cancer therapy in combination with mitophagy inhibitors. These mito-toxic antibiotics inhibit mitochondrial protein translation, similar to their action in bacteria, resulting in a ‘mitonuclear’ protein imbalance that activates the mitochondrial unfolded protein response (UPR^mt^) that is commonly resolved by mitophagy [50,123]. Treatment of tumor cells with any one of these drugs would be predicted to elicit an acute dependence on mitophagy for survival before other adaptive survival mechanisms come into play. Thus, combining one or more of these drugs with a drug that inhibits mitophagy may provide added benefit in terms of treating cancers.

## Conclusions

Mitophagy is a clearly distinct form of autophagy involving the selective degradation of mitochondria at the autophagolysosome. Specific defects in mitophagy have been linked to human cancers through deletion of key regulators such as Parkin and BNIP3. Additionally, mouse models reveal distinct phenotypes when mitophagy is specifically inhibited compared to that observed when general autophagy is inhibited. Targeting mitophagy may therefore offer opportunities to more selectively inhibit tumor progression to malignancy where one may take advantage of the acute sensitivity of tumor cells to mitochondrial dysfunction when combined with other drugs or stresses.

## Abbreviations

AMPK: AMP (adenosine monophosphate)-regulated kinase
ATP: adenosine triphosphate
Bcl-2: B-cell leukemia/lymphoma protein-2
BH3: Bcl-2 homology domain 3
BNIP3: Bcl-2/adenovirus E1B interacting protein-3
BNIP3L: BNIP3-like
DCIS: ductal carcinoma in situ
FIP200: FAK interacting protein 200
FUNDC1: FUN14 domain containing 1
HDAC: histone deacetylase
HIF: hypoxia-inducible factor
IMM: inner mitochondrial membrane
IMS: intermembrane space
LC3: light chain 3
LIR: LC3-interacting region
Mcl-1: myeloid cell leukemia-1
Mfn-2: Mitofusin-2
mtDNA: mitochondrial DNA
MTs: microtubules
NBR1: neighbor of BRCA1
NIX: Nip-like protein-X
OPA-1: optic atrophy-1
OMM: outer mitochondrial membrane
OXPHOS: oxidative phosphorylation
PINK1: PTEN-induced putative kinase-1
PKA: protein kinase A
RB: retinoblastoma tumor suppressor
RB1CC1: RB1-inducible coiled coil 1
ROS: reactive oxygen species
SQSTM1: sequestosome-1
TNBC: triple negative breast cancer
ULK-1: unc-51 like autophagy activating kinase-1
VDAC: voltage-dependent anion channel
